# Supplementary material for: Iterative improvement in the automatic modular design of robot swarms
Source: PeerJ Comput Sci. 2020 Dec 7;6:e322. doi: 10.7717/peerj-cs.322 (PMC7924708; doi:10.7717/peerj-cs.322)
Supplement: Supplemental Information 3 [file peerj-cs-06-322-s003.zip › argos3/doc/api/standalone/a00616_source.html]

ARGoS: plugins/simulator/physics\_engines/dynamics2d/dynamics2d\_stretchable\_object\_model.h Source File


- Main Page
- Related Pages
- Namespaces
- Classes
- Files

- File List
- File Members

# plugins/simulator/physics\_engines/dynamics2d/dynamics2d\_stretchable\_object\_model.h

Go to the documentation of this file.

```
00001 
00007 #ifndef DYNAMICS2D_STRETCHABLE_OBJECT_MODEL_H
00008 #define DYNAMICS2D_STRETCHABLE_OBJECT_MODEL_H
00009 
00010 namespace argos {
00011    class CDynamics2DGrippable;
00012    class CDynamics2DStretchableObjectModel;
00013 }
00014 
00015 #include <argos3/plugins/simulator/physics_engines/dynamics2d/dynamics2d_single_body_object_model.h>
00016 
00017 namespace argos {
00018 
00019    /****************************************/
00020    /****************************************/
00021 
00032    class CDynamics2DStretchableObjectModel : public CDynamics2DSingleBodyObjectModel {
00033 
00034    public:
00035 
00036       CDynamics2DStretchableObjectModel(CDynamics2DEngine& c_engine,
00037                                         CComposableEntity& c_entity);
00038 
00039       virtual ~CDynamics2DStretchableObjectModel();
00040 
00041       virtual void MoveTo(const CVector3& c_position,
00042                           const CQuaternion& c_orientation);
00043 
00044       virtual void Reset();
00045 
00046       virtual void UpdateFromEntityStatus() {}
00047 
00048       inline Real GetMass() const {
00049          return m_fMass;
00050       }
00051 
00052       inline void SetMass(Real f_mass) {
00053          m_fMass = f_mass;
00054       }
00055 
00056       inline void SetGrippable(CDynamics2DGrippable* pc_grippable) {
00057          m_pcGrippable = pc_grippable;
00058       }
00059       
00064       void SetLinearFriction(Real f_max_bias,
00065                              Real f_max_force);
00066 
00071       void SetAngularFriction(Real f_max_bias,
00072                               Real f_max_force);
00073 
00074    private:
00075 
00076       Real m_fMass;
00077       CDynamics2DGrippable* m_pcGrippable;
00078       cpConstraint*         m_ptLinearFriction;
00079       cpConstraint*         m_ptAngularFriction;
00080    };
00081 
00082    /****************************************/
00083    /****************************************/
00084 
00085 }
00086 
00087 #endif
```

---

Generated on 10 Jul 2018 for ARGoS by 
 1.6.1
